# Psychedelics: A new era of treatment?

**DOI:** 10.1192/j.eurpsy.2021.1290

**Published:** 2021-08-13

**Authors:** S. Torres

**Affiliations:** Psychiatry And Mental Health Department, Centro Hospitalar Barreiro-Montijo, Barreiro, Portugal

## Abstract

**Introduction:**

Psychedelics - including LSD (lysergic acid diethylamide), psilocybin, DMT (N, N-dimethyltryptamine), ayahuasca and mescaline - have an ancient history across various civilizations. In 1950, after LSD’s discovery by Hofmann, psychedelics enjoyed a short-lived relationship with psychiatry, before prohibitive legislature emerging in response to the recreational use in the mid-1960s. However, the last decade has witnessed a renewed scientific interest in psychedelics - a phenomenon referred to as the ‘Psychedelic Renaissance’.

**Objectives:**

Review the pharmacology of psychedelic drugs and the latest evidence of its therapeutic potentials in anxiety, mood and addictive disorders.

**Methods:**

Literature review performed on PubMed and Google Scholar databases, using the keywords “psychedelics”, “hallucinogens”, “d-lysergic acid diethylamide (LSD)”, “psilocybin”, “ayahuasca”, “mescaline”, “DMT (N,N-dimethyltryptamine)”.

**Results:**

The psychedelics or “classic hallucinogens” can be subdivided into three sub-classes: the plant-derived tryptamines (psilocybin and ibogaine) and phenethylamines (mescaline), and the semisynthetic ergolines (LSD). The therapeutic potentials are mediated by an agonist action on 5-HT2A receptors expressed in frontal and paralimbic structures involved in mood and emotion regulation, introspection, interoception and self-consciousness. Stimulation of 5-HT2ARincreases the glutamatergic tone and neuroplasticity and is accompanied by reduced amygdala activity, reducing anxiety. Experimental, open-label, and RCTs showed anxiolytic, antidepressive, and antiaddictive effects with psychedelics. As examples, psilocybin and LSD reduced anxiety and depression in cancer patients and symptoms of alcohol and tobacco dependence, and ayahuasca reduced depression in treatment-resistant depression.

**Conclusions:**

Despite the promising effects of psychedelics on anxiety, depression and addiction, the evidence is still preliminary, waiting for long-term studies with bigger samples.

**Conflict of interest:**

No significant relationships.

